# Author Correction: Reversible two-way tuning of thermal conductivity in an end-linked star-shaped thermoset

**DOI:** 10.1038/s41467-024-51527-y

**Published:** 2024-08-15

**Authors:** Chase M. Hartquist, Buxuan Li, James H. Zhang, Zhaohan Yu, Guangxin Lv, Jungwoo Shin, Svetlana V. Boriskina, Gang Chen, Xuanhe Zhao, Shaoting Lin

**Affiliations:** 1 Department of Mechanical Engineering, Massachusetts Institute of Technology, Cambridge, MA USA; 2 Department of Mechanical Engineering, Michigan State University, East Lansing, MI USA; 3 Department of Civil and Environmental Engineering, Massachusetts Institute of Technology, Cambridge, MA USA

**Keywords:** Polymers, Mechanical engineering

Correction to: *Nature Communications* 10.1038/s41467-024-49354-2, published online 03 July 2024

The original version of the Supplementary Information associated with this Article contained an error in author list. The correct version of author list is: Chase M. Hartquist, Buxuan Li, James H. Zhang, Zhaohan Yu, Guangxin Lv, Jungwoo Shin, Svetlana V. Boriskina, Gang Chen, Xuanhe Zhao & Shaoting Lin, which replaces the previous incorrect version: Shaoting Lin, Chase Hartquist, Buxuan Li, James H. Zhang, Zhaohan Yu, Guangxin Lv, Jungwoo Shin, Svetlana V. Boriskina, Gang Chen, Xuanhe Zhao. The HTML has been updated to include a corrected version of the Supplementary Information.

### Supplementary Information

Revised Supplementary Information

