# Supplementary material for: Reversible two-way tuning of thermal conductivity in an end-linked star-shaped thermoset
Source: Nat Commun. 2024 Jul 3;15:5590. doi: 10.1038/s41467-024-49354-2 (PMC11222444; doi:10.1038/s41467-024-49354-2)
Supplement: Supplementary file 1 — Supplementary Information [file 41467_2024_49354_MOESM1_ESM.pdf]

# Reversible Two-way Tuning of Thermal Conductivity in an End-linked Star-shaped Thermoset

Chase M. Hartquist[1, 4], Buxuan Li[1, 4], James H. Zhang[1, 4], Zhaohan Yu[2], Guangxin Lv[1], Jungwoo Shin[1], Svetlana V. Boriskina[1], Gang Chen[1*], Xuanhe Zhao[1, 3*], Shaoting Lin[2, 3*]

**Affiliations:**

[1]Department of Mechanical Engineering, Massachusetts Institute of Technology, Cambridge, MA, USA.

[2]Department of Mechanical Engineering, Michigan State University, East Lansing, MI, USA

[3]Department of Civil and Environmental Engineering, Massachusetts Institute of Technology, Cambridge, MA, USA.

[4]These authors contribute equally: Chase M. Hartquist, Buxuan Li, James H. Zhang

*Corresponding author. Email: gchen2@mit.edu, zhaox@mit.edu, linshaot@msu.edu

**This PDF file includes:**

Materials and Methods

Supplementary Text

Table S1-S4

Figs. S1-S20

References

## 1. Material synthesis and sample preparation

**Materials.** 20,000 MW tetra-arm amine-terminated PEG (Laysan Bio, 4 arm PEG-$NH_2$, MW 20,000) and 20,000 MW tetra-arm NHS-terminated PEG (Laysan Bio, 4 arm PEG-SG, MW 20,000) were the two types of macromers used for synthesizing 20,000 MW ELST. Phosphate buffered saline (Sigma-Aldrich, P4417) was used as the buffer to dissolve amine-terminated PEG, and a phosphate-citrate buffer (Sigma-Aldrich, P4417) was used to dissolve SG-terminated PEG. In preparing the sample for FDTR thermal conductivity measurement, the EMbed 812 Embedding Kit (Electron Microscopy Sciences) was used to embed ideal-network polymers in preparation for microtome cutting.

**Synthesis of the ELST.** We started by synthesizing the ideal-network PEG hydrogel reported in past work [1, 2]. 100 mg of tetra-arm amine-terminated PEG was first dissolved and vigorously mixed in a 1 mL phosphate buffer solution (one tablet dissolved in 300 mL deionized water), yielding a pH of 7.4 and ionic strength of 100 mM. Thereafter, 100 mg of tetra-arm amine-terminated PEG was dissolved and vigorously mixed in 1 mL of a phosphate-citrate buffer solution (one tablet dissolved in 150 mL deionized water), yielding a pH of 5.8 and ionic strength of 100 mM. Both the solutions of PEG-$NH_2$ and PEG-NHS were vigorously mixed and poured into an acrylic mold, giving a final concentration of 50 mg/mL for both PEG-$NH_2$ and PEG-NHS macromers. The resultant samples were placed in a humidity chamber for at least 12 hours to complete the reaction of forming amide bonds between macromers. To obtain the solvent-free tetra-PEG thermoset, the as-prepared tetra-PEG hydrogel was placed in an environmental chamber at 37 °C for subsequent dehydration. To enable uniform and isotropic shrinkage of ideal-network PEG hydrogels, a thin layer of silicon oil was introduced at the interface between the hydrogel and the substrate to mitigate interfacial adhesion. The complete dehydration process typically took about 12 hours. Samples were carefully heated above $T_m$ and cooled to room temperature to alleviate any residual stress buildup.

**Preparation of the ELST with controlled stretch ratios.** To further prepare tetra-PEG thermosets with controlled stretch ratios (**Fig. S2a**), we placed the dried ideal-network PEG polymers at stress-free state in an oil bath at an elevated temperature of 50 °C and further used a mechanical tester (CellScale, Canada) to uniaxially stretch the sample up to a controlled stretch ratio $\lambda^*$. At the controlled stretch ratio, the stretched sample was removed from the oil bath for cooling. The cooling process took around 1 hour for a sample with thickness of around 1 mm. After sufficient cooling, the chain configuration – including crystalline orientation and amorphous chain alignment – were well preserved in the sample when subject to controlled stretch ratios. To examine the durability of the sample for preserving giant reversible tuning of thermal conductivity, we also prepared the tetra-PEG thermosets after cyclic loading at a controlled stretch ratio of 10.

**Preparation of the ELST for FDTR thermal measurements.** The FDTR thermal measurements require ultra-smooth sample surfaces for achieving a desirable reflectance ratio. We embedded the PEG sample in an epoxy resin exposing the sample's cross-section as the top surface for subsequent surface polishing. The pre-gel epoxy resin was prepared by mixing 13 ml of solution A (prepared by mixing 20 mL EMBed-812 and 31 mL DDSA), 15 ml of solution B (prepared by mixing 20 mL EMBed-812 and 17 mL NMA), and 500 μL of DMP-30 at room temperature

overnight. EMBed-812, DDSA, NMA, and DMP-30 are provided in the Embed 812 Embedding Kit (Electron Microscopy Sciences). To improve the quality of the epoxy, we degassed the epoxy resin with the sample embedded for about 90 minutes, followed by a subsequent curing in the ambient environment overnight. The top surface of the sample embedded in epoxy resin was cyclically cut using a microtome machine (Leica EM UC7 ultramicrotome) with a glass knife. The feeding rate was carefully selected with a relatively high rate initially 5.00 mm/s for facing and reduced incrementally to 1.00 mm/s for a final cut. The feed was initially set as 300 nm and then reduced incrementally to 50 nm for the final cut. All decisions for changing parameters were based on surface/quality/conditions as viewed by the microscope. A new glass knife was used for the final 3 to 5 passes to improve quality. The surface-polished sample was stored in a plastic bag to prevent deliquescence of PEG polymers, which might affect the surface quality over time.

## 2. Steady-state Differential Thermal Conductivity Stage

We also used a home-built steady state differential thermal conductivity stage (**Fig. S3a**) to measure the thermal conductivity of samples along the stretching direction. The stage has been extensively validated [3]. In previous work [4], this platform has been successfully used to measure reference samples such as ultrathin polyethylene films, 304-stainless steel foils, Dyneema fibers, Zylon fibers, Sn and Al films, with thermal conductive range from 0.38 W m$^{-1}$ K$^{-1}$ (unstretched polyethylene film), to 202.7 W m$^{-1}$ K$^{-1}$ (Al films).

**Principle.** Using the steady-state system, we measured the time-invariant heat flux given a set of constant temperature differences across the sample. The sample was mounted between a hot junction and a cold junction, which were connected to a temperature-controlled heater and a thermoelectric cooler (TEC), respectively. The TEC dissipates heat to a water-cooled heat sink. Thermocouples were connected to the heater and a cold junction, whose temperatures were measured. The heat flux measured as the electrical heating power of heater ($P_e$) was monitored by measuring the imposed voltage and current. The platform was surrounded by a copper radiation shield, whose temperature was measured by a thermocouple and controlled at the same temperature as the hot junction via a heater. The radiation shield was thermally insulated from the water-cooled heat sink by cylindrical porous ceramic spacers. The whole platform was put into a vacuum chamber. By maintaining the temperature of the radiation shield at that of the hot junction, the radiative loss to the environment was minimized and negative; neglecting this value yields a conservative result that underestimates the thermal conductivity of samples. The measurement was performed once a steady state temperature difference (less than 0.1 K standard deviation over 1 minute at a sampling frequency 1 Hz) and ample vacuum (less than 5e$^{-6}$ mbar with a turbomolecular pump) were established. In the platform, the one-dimensional Fourier law of heat conduction was satisfied for film shaped samples. Given the width $w$, length $L$, and thickness $t$ of a sample, the heat flux across sample ($Q$) given a temperature difference between the hot and cold junctions can be expressed as:

$$Q = \frac{\kappa w t}{L}(T_h - T_c) \tag{S1}$$

With the determination of sample geometry, temperature difference, and heat flux, we used the Fourier law of heat conduction above to calculate the sample thermal conductivity $\kappa$.

**Parasitic heat loss minimization and radiative contribution correction.** Although we have minimized the convection and radiative heat loss from the sample to the environment, the heater power input is different from the heat flux across samples due to parasitic heat loss through the electric component of the heater. We minimized the parasitic heat loss by measuring the differential heat flux at varying temperature differences. We measured the heat flux under 5 temperature differences, namely 2, 4, 6, 8, and 10 K, where the heater temperature is kept at 300 K and the TEC temperature changes accordingly. A linear regression between the measured input power and temperature difference was performed to extract the thermal conductance $\frac{kwt}{L}$: $P_e = \frac{kwt}{L}\Delta T + \text{const.}$ The advantages of such differential measurement are twofold. Firstly, parasitic heat loss is dominant at the hot junction, which is kept at a constant temperature; therefore, parasitic heat loss can be assumed as a constant. Secondly, while systematic error exists for their absolute temperature measurement, thermocouples are accurate in measuring the temperature changes. As such, the measurement error of thermocouples at both junctions, and most of the parasitic heat losses, will be lumped into the interception constant, and therefore would not affect the slope of the regression. The radiative loss was carefully corrected by performing a two-step measurement, where in the second measurement the sample was cut in half along its midline and the view factor between the hot and cold junction remains unchanged, as shown in **Fig. S3b-e**. The difference of measured thermal conductivity is the contribution from the film sample without direct radiative transfer between two junctions.

**Identification of thermal conductivity.** As shown in **Fig. S3f**, the temperature was sampled every second and a steady state temperature difference was clearly established at every measurement. The heat power linearly depends on the temperature difference and the fitted thermal conductivity of a 20,000 MW sample with stretch ratio of 8 was measured to be 1.04 W $m^{-1}$ $K^{-1}$.

## 3. Frequency Domain Thermoreflectance (FDTR)

We used a frequency domain thermoreflectance (FDTR) to measure the thermal conductivity of ideal-network PEG polymers, following the method developed by Schmidt et al [5-8].

**Sample preparation.** The stretched and unstretched samples were embedded in a low-temperature epoxy (EMBed-812). The epoxy embedded sample was vacuumed to remove any air bubbles and cured (**Fig. S5a**). A rough surface perpendicular to the stretching direction was cut to expose the sample surface using a razor blade (**Fig. S5b**). Then the exposed surface was carefully cut using a microtome machine (Leica EM UC7 ultramicrotome). The cut exposed surface (**Fig. S5c**) was then coated with 200-nm Au films by DC-magnetron sputter (Leica EM ACE600). The Au film served as an optical transducer for FDTR measurements.

**Laser parameters and mechanism.** The FDTR platform employed a continuous wave (CW) pump laser with a wavelength of 488 nm, and another CW probe laser with a wavelength of 532 nm. The pump laser was sinusoidally modulated from around 3 kHz to 10 MHz. The $1/e^2$ diameter of the laser spot on the sample was about 4.0 µm for the pump. The pump sinusoidally heated the sample while the probe sensed the modulated sample surface temperature via the thermoreflectance effect of the coated transducer layer. As illustrated in **Fig. S5d**, the power of the reflected probe beam was detected by a balanced detector, using a reference beam split from the

laser source to minimize the noise due to the laser power fluctuation. The output of the balanced detector was given to a radio-frequency lock-in amplifier. Before sample measurement, the phase of the pump beam at each modulation frequency was predetermined. The phase lag between the modulated surface temperature and the sinusoidal heating was then recorded as the FDTR signal. The measured FDTR phase data was fitted with an isotropic two-layer analytical model using the sample thermal conductivity and the Au-sample interfacial conductance as fitting parameters [5-8]. The isotropic two-layer analytical model requires the sample's volumetric heat capacity as an input, which was identified as 1.67 $e^{-6}$ J $m^{-3}$ $K^{-1}$ (equivalently 29,604 J $mol^{-1}$ $K^{-1}$) for 20,000 MW PEG at T = 300 K [9]. While anisotropic thermal conductivity is expected for stretched samples, the difference between thermal conductivities in out-of-plane (along the stretching) and in-plane (perpendicular to the stretching) directions are neglected. In FDTR, the pump and probe laser spots are well aligned and overlapped with zero offset; therefore, a one-dimensional transient heat conduction along the out-of-plane direction is assumed in the model, leaving the measured signal negligible sensitivity on the in-plane thermal conductivity.

**Identification of thermal conductivity and interfacial conductance.** The measured phase lag from 3 kHz to 10 MHz was fitted using the Monte Carlo method with 1000 random inputs. The thermal conductivity along the stretching direction, and the interfacial thermal conductance between sample and the coated Au film, were fitted. The best fitted curve and another two curves with ± 10% variation of thermal conductivity identified the best fitted value. We measured a series of samples with a stretch ratio from 1 (unstretched) to 15. We also demonstrated the thermal conductivity of stretched and released sample at stretch ratio of 15 for up to 500 stretching cycles. **Figure S5e** plots a representative fitted curve, where the thermal conductivity is identified as 1.11 W $m^{-1}$ $K^{-1}$ and the thermal conductance between sample and Au film is identified as 7.9 × $10^7$ W/K.

## 4. Photoelasticimetry Experiments

We designed a photoelasticimetry experimental setup to measure the internal stress of the polymer based on the observed changes in light intensity resulting from alternations in its molecule structure. As illustrated in **Fig. S9a**, the photoelasticimetry experimental setup consists of a light source, two linear polarizers, two quarter wave plates, a universal mechanical tester, and a camera. When a material undergoes mechanical strain, the material exhibits a visual pattern of fringes, referred to as photoelastic response. This fringe pattern correlates with the internal stress associated with the molecule structure.

Specifically, we performed relaxation photoelasticimetry experiments on our ELST (**Fig. S10a, b**), measuring the nominal stress of the material $s$ and the intensity at specific locations on the material $I$ as a function of time $t$. As illustrated in **Fig. S9b-d**, by fitting the measured $S(t)$ and $I(t)$ into the Voigt model [10], we can extract the mean response time for the structural change of the entire material $\tau_m$ and the response time for the structural change at specific locations on the material $\tau$ via the following equations, $\frac{S(t)-S_{min}}{S_{max}-S_{min}} = 1 - e^{t/\tau_m}$, $\frac{I(t)-I_{min}}{I_{max}-I_{min}} = 1 - e^{t/\tau}$, where $S_{max}$ and $S_{min}$ are the maximum and minimum nominal stress of the material, $I_{max}$ and $I_{min}$ are the maximum and minimum intensity at specific locations on the material. **Figure S10c** shows a representative force of our ELST as a function of time, measuring $\tau_m$; and **Figure S10d** presents representative intensities at specific locations of our ELST as a function of time, measuring corresponding $\tau$ at

these locations. The value of $\tau_m$ and the mean value of spatially distributed $\tau$ are consistently on the order of 1 second, which indicates the response time for structure change in the sample is almost the same as the response time for stress relaxation of the entire sample. Physically, these two response times align with the response time for the thermal conductivity tuning since the polymer's thermal conductivity is inherently linked to its molecular structure.

To increase light intensity – which improves measurement accuracy – through amplified stress levels, we introduced a crack at the center of the sample for inducing stress concentration. While the introduction of the crack can enhance resolution, the presence of a crack might potentially induce experimental error due to the altered stress state in the material. To examine the accuracy of the experimental results due to the different stress levels applied on the material, we plot the intensity versus time at different locations away from crack tip. As shown in **Fig. S10d**, the extracted relaxation time at different locations is consistent on the order of 1 s.

It should be noted that the relaxation photoelasticimetry experiment has two limitations. The first limitation lies in its indirect measurement nature, introducing potential sources of errors. For example, the resolution of the measured response time is dependent on the optical system's ability to detect the color change of our ELST. The second limitation is that this technique only works for optically transparent or translucent materials. Fortunately, our ELST becomes optically transparent when heated above its melting temperature, allowing us to effectively utilize the relaxation photoelasticimetry experiment.

We specifically perform comparison photoelasticimetry experiments on the end-linking star-shaped thermoset (ELST) and a polyacrylamide-glycerol hydrogel (PAAm) representing a conventional polymer, demonstrating a much-reduced response time for structural alternation in the ELST.  As illustrated in **Fig. S10a**, both the ELST and the PAAm are subjected to an instantaneous stretch (i.e., stretch of 4.4 in 1 second for the ELST, and stretch of 1.8 in 1 second for the PAAm). As shown in **Fig. S10e**, the response time for structural alternation of the PAAm sample is around 100 s; in contrast, the response time for structural alternation of the ELST sample is 2.58 s, orders of magnitude shorter than that of the PAAm. The short response time for structural alternation in the ELST serves as indirect evidence to substantiate the claim of rapid thermal conductivity tuning in the ELST.

## 5. Small- and Wide- angle X-ray Scattering

Small-angle X-ray scattering (SAXS) and wide-angle X-ray scattering (WAXS) were performed using a Dectris Pilatus3R 300K detector on a SAXSLAB apparatus (X-ray Diffraction Shared Experimental Facility at Massachusetts Institute of Technology). The vacuum chamber was pumped to 0.08 mbar during measurements to reduce background intensity fluctuation. Details on measurement configurations are listed in **Table S2**. Tensile specimens (~ 4 mm × 1 mm × 0.6 mm) were stretched for bulk structural characterization using the same procedure described for thermal conductivity measurement prior to X-ray measurement. Tetra-PEG thermosets were cooled to room temperature and fixed on either end with Krazy glue to an acrylic mount.

To identify crystallinity, the 2D SAXS and WAXS scans were converted to 1D intensity profiles by averaging over all azimuthal angles. Although some reported crystallinity measurements average over a small azimuthal range, we counter that this strategy causes inflated crystallinity

measurements in anisotropic samples. The average intensity measured during scattering was plotted against the scattering angle $2\theta$, which relates to the scattering vector $q$ as follows:

$$q = \frac{4\pi\, sin(2\theta/2)}{\Lambda} \tag{S2}$$

where $\Lambda$ is the X-ray wavelength.

This quantity relates to the characteristic interplanar d-spacing through Bragg's law, which is defined as:

$$2d\, sin\, \theta = n\Lambda \tag{S3}$$

where $d$ is the interplanar spacing and $n$ is the diffraction order.

Diffraction spots were indexed according to their d-spacing and the associated crystal lattice configuration.

$$d_{hkl} = \frac{2\pi}{h\vec{\boldsymbol{a}} + k\vec{\boldsymbol{b}} + \boldsymbol{l}\vec{\boldsymbol{c}}} \tag{S4}$$

where $h$, $k$, and $l$ are Miller indices, and $\vec{\boldsymbol{a}}$, $\vec{\boldsymbol{b}}$, and $\vec{\boldsymbol{c}}$ are the basis of vectors for the reciprocal lattice [11-13].

Spacing between repeating characteristic structural features ($L$) was determined by the $d$-spacing of the first distinct intensity peak of the 1D scattering intensity from SAXS scans averaged about azimuthal angles, $\phi$ = 90 ± 5 degrees.

The crystallinity index was evaluated by fitting Gaussian or Pseudo-Voigt curves to the amorphous and crystalline peaks distinguished from the 1D intensity profile after subtracting the background scattering intensity. Summing the areas under the fitted curves enables deduction of the crystallinity index as follows:

$$\chi = \frac{A_C}{A_C + A_A} \tag{S5}$$

where $A_C$ is the area under the crystalline peaks and $A_A$ is the area under amorphous peaks (**Fig. S16**).

The orientation of the crystalline regime was determined from the azimuthal spread of intensity at the $d$-spacing corresponding to a given diffraction peak (**Fig. S17**) [14]. Orientation was defined for a peak at given Miller indices from a 2D WAXS pattern as follows:

$$\langle \cos^2\phi \rangle_{hkl} = \frac{\int_0^{\pi/2} I(\phi)\cos^2\phi \sin\phi \, d\phi}{\int_0^{\pi/2} I(\phi) \sin\phi \, d\phi} \tag{S6}$$

where $I(\phi)$ is the intensity as a function of the azimuthal angle $\phi$.

The Hermans orientation parameter $f_2$ was determined from this orientation measure as follows:

$$f_2 = \frac{3\langle \cos^2\phi \rangle - 1}{2} \tag{S7}$$

where $f_2$ takes the value -0.5 when the crystal is aligned perpendicular to the direction of interest, 0 when there is no preferred direction, and 1 when aligned parallel to the reference direction [15].

The Scherrer equation [15] was applied to the 1D peak fitting scheme to evaluate effective crystallite sizes.

$$D = \frac{K\Lambda}{B(2\theta)\cos\theta} \tag{S8}$$

where $D$ is the crystallite size, $K$ is the Scherrer constant or shape factor ($K$ = 0.94 for full width at half maximum or $FWHM$ measurements), $\Lambda$ is the wavelength of the X-ray, $B$ is the breadth or $FWHM$ of the fit profile to the peak at a given $2\theta$, and $\theta$ is the Bragg angle associated with the peak of interest.

## 6. Full-atom Molecular Dynamics Simulations

**Simulation Setup.** This work used all-atom molecular dynamic (MD) simulations to model the thermal conductivity of mechanically strained PEG ideal networks. The COMPASS force field was used for these simulations. COMPASS is a class II force field parametrized for organic molecules, inorganic molecules, and polymers [16, 17]. The COMPASS force field has been successfully used to study both thermal transport and mechanical properties in polymer systems due to its accurate parametrization of macroscopic properties, conformational energies, and molecular vibrations. The cut-off distance for pair interactions was set at 10 Å. Long-range electrostatic interactions are calculated using the particle-particle particle-mesh PPPM algorithm, and Lennard-Jones tail corrections are included. Following the original parametrization of the COMPASS force field for PEG units, a background dielectric constant of 1.4 was used [17].

**Figure S18** illustrates the detailed procedures for sample initialization, stretching and equilibration, and production steps. A fully extended, 1 × 2 × 2 diamond lattice of the ideal polymer network was initialized in the simulation cell. Each 4 arm PEG macromolecule has a molecular weight of 10k g/mol, causing each of the macromolecule's arms to have 57 ether units. A short energy minimization run was conducted for a maximum of 1000 steps using the conjugate gradient algorithm followed by a constant NVT (canonical ensemble, constant particle number, simulation domain volume, and temperature) run at 596 K for 10 ps to remove high energy configurations. For this step, the timestep was set to 0.1 fs and the Berendsen thermostat with a time constant of 10 fs was used to ensure stability. Next, the thermostat was switched to the Langevin thermostat with a time constant of 100 fs and the timestep was increased to 1 fs. The polymer networks were compressed at a constant rate of 0.5 nm/fs in each axis to about ¼ of the final density at room temperature. This step was expedited by excluding long range electrostatic interactions due to the low initial density of the system, with the fully extended diamond lattice having an initial density of about 0.1 mg/cm$^3$.

Afterwards, the timestep was set to 0.5 fs and long-range electrostatic interactions were included in all subsequent steps. An annealing series of NVT runs for 200 fs and NPT runs for 200 fs at 1 atm were conducted for 5 loops to equilibrate the system density. After the annealing steps, the system was equilibrated for an additional 10 ns in the NPT ensemble. The Berendsen barostat was used with a time constant of 10 ps, and a Langevin thermostat was used with a time constant of

0.1 ps. The high initial time constant used in the barostat was selected to decrease the compression rate of the simulation cell.

Afterwards, another NPT run was conducted for 5 ns with the Berendsen time constant reduced to 1 ps. A trajectory snapshot was outputted every 500 ps to create an independent initial condition for subsequent stretching simulations. Each of the snapshots were then cooled at a rate of 20 K/ns to 298 K and equilibrated at 298 K for 5 ns. The system average room temperature density of 1.129 g/cm$^3$ was calculated by averaging the densities of the final 1 ns of all runs during this equilibration step, which is very close to experimental densities of the PEG at room temperature.

To generate the stretched samples, the thermostat was first switched to the Nosé-Hoover thermostat with a time constant of 100 fs. The volumes of all simulation cells were initially adjusted to match the system average density over 100 fs. A Parrinello barostat was then coupled to the y and z-directions while keeping the x-direction fixed. The y and z-directions were coupled in the barostat calculations and the barostat's time constant was set at 1 ps. The system was heated to 353 K at a rate of 20 K/ns and then equilibrated for 1 ns. The samples were then strained along the x-direction at a constant engineering strain rate of 0.5 ns$^{-1}$. Snapshots were periodically output to generate samples at different stretch ratios. Each of the snapshots were then cooled to 298K at a rate of 20 K/ns and equilibrated for 5 ns. The average density for each stretch ratio was calculated over the last 1 ns of the equilibration run. The system was switched to a NVT ensemble and each of the sample's y and z-direction lengths were changed over 100 ps to match the average density values. A subsequent 5 ns equilibration run was conducted again to relax the system after the density change. Finally, each of the samples were switched to a NVE ensemble to calculate its structure and thermal conductivity properties over a 1 ns simulation time.

Due to the low convergence for the 10x stretch ratio thermal conductivity, additional samples were generated using the following protocol. After the stretched samples were cooled to 298 K, each of the samples were equilibrated for an additional 200 ps in the NVT ensemble at 353 K and a snapshot was outputted every 100 ps, leading to a total of 27 generated samples. All these samples were then used for the NVE production runs for the 10x stretch ratio samples.

The ideal network displays a higher order structure due to its regular diamond structure as visualized in **Fig. 6a**. We calculated the radial distribution function (RDF) curves of the crosslinker atoms, which show distinct peaks at different distances due to its tetrahedral structure as shown by the blue curve in **Fig. S19**. Once the sample is stretched in an axis, the first distinct peak separates into three smaller peaks due to the restructuring of the molecular topology.

**Calculation of Order Parameters.** The structures of the different samples were calculated to understand the effects of crystallinity and polymer chain alignment on the material's thermal conductivity. Based on the $p_2$ order parameter, two different order parameters are calculated.

$$p_{2,x} = \langle \frac{3}{2}\cos^2\theta_{i,x} - \frac{1}{2} \rangle_i \qquad \text{(S9-1)}$$

$$p_{2,\text{global}} = \langle \frac{3}{2}\cos^2\theta_{i,j} - \frac{1}{2} \rangle_{ij} \qquad \text{(S9-2)}$$

where $\theta$ is the angle between two vectors, $i$ represents the vector connecting the $(i-1)$ atom to the $(i+1)$ atom along the chain backbone, $j$ represents the vector connecting the $(j-1)$ atom to the (j

+ 1) atom along the chain backbone, and $x$ represents the cartesian axis along the stretch direction. $p_{2,global}$ can give an understanding of the overall crystallinity of the simulation cell at different stretch ratios and $p_{2,x}$ describes the overall chain alignment along the stretch direction. 50 snapshots were generated for each independent simulation and averaged for each stretch ratio during the calculation.

**Thermal Conductivity Methods.** The thermal conductivity of each sample, $\kappa$, was calculated using the Green-Kubo method. The heat flux vectors, **J**, were calculated at every timestep as

$$\kappa = \frac{V}{k_B T^2} \int_0^\infty \langle \mathbf{J}(t)\mathbf{J}(0) \rangle dt \tag{S10}$$

where $\mathbf{J}(t)$ is the heat flux vector at a given time $t$, $V$ is the system volume, and $T$ is the system temperature. The heat flux vector can be decomposed into a convective term and a virial term.

$$\kappa = \kappa_{\text{conv}} + \kappa_{\text{vir}} \tag{S11}$$

$$\kappa_{\text{virial}} = \kappa_{\text{nonval}} + \kappa_{\text{val}} \tag{S12}$$

The virial term can be decomposed further into contributions from non-valence and valence terms. The valence terms are calculated using the proposed centroid calculation that correctly considers the effects of many body interactions in polymer systems [18-20]. The valence terms include contributions from two-body bonds, three-body angles, four-body dihedral, four-body improper interaction terms, and cross-correlation terms between the prior four terms as described by the COMPASS force field.

Thus, the total thermal conductivity can be decomposed into four contributions: convection autocorrelation, non-valence autocorrelation, valence autocorrelation, and cross-correlation terms, namely

$$\kappa = \kappa_{\text{conv-conv}} + \kappa_{\text{nonval-nonval}} + \kappa_{\text{val-val}} + \kappa_{\text{cross}} \tag{S13}$$

It was found that the Green-Kubo correlations plateaued at around 10 ps, so the integral was cutoff at this mark (**Fig. S20**). **Figure 6b** plots the four contributions to the total thermal conductivity. It is primarily the valence-valence correlation term that leads to enhanced thermal conductivity with stretch ratio. The convection and non-bonded terms stay relatively constant throughout the simulation while the cross correlations show some more variations.

# Table S1. Comparison between the ELST and existing thermal switches

| Materials | Mechanism | Polymer | Tunin g ratio | Response time (s) | Continuous | Reversibility | Size | Cycles | Ref |
|---|---|---|---|---|---|---|---|---|---|
| $SrCoO_{2.5}$ | Electrochemical intercalation | No | 2.5 | 300 | Yes | Yes | 27-44 nm thickness | 2 | [21] |
| $MoS_2$ | Electrochemical intercalation | No | 10 | ~ 200 | Yes | Yes | ~ 10 nm thickness | 2 | [22] |
| Lithium cobalt oxide | Electrochemical lithiation | No | 1.46 | ~ 3600 | Yes | Yes | 497 nm thickness | 2 | [23] |
| Graphene nanoparticle | Electrical field | No | 1.4 | 14 | Yes | Yes | / | 4 | [24] |
| Graphite nanoflake | Magnetic field | No | 3.25 | / | Yes | Yes | / | 5 | [25] |
| VO2 doped with tungsten | Metal-insulator transition | No | 1.5 | / | No | / | ~ 10 µm length<br>~ 100 nm diameter | / | [26] |
| Graphite/ hexadecane | Solid-liquid transition | No | 3.2 | / | No | / | / | / | [27] |
| CNT/ hexadecane | Solid-liquid transition | No | 3.0 | / | No | / | / | / | [28] |
| Lead zirconate titanate (PZT) | Domain polarization by electrical field | No | 1.2 | $4\times10^{-8}$ | No | Yes | ~ 100 nm thickness | 5 | [29] |
| Bismuth-antimonide alloy | Domain polarization by magnetic field | No | 1.2 | / | No | / | 1-20 µm thickness | / | [30] |
| Carboranethiol cage molecules | Self-assembled molecular junctions | No | 13.4 | $1.5\times10^{-7}$ | Yes | Yes | Monolayer | $10^6$ | [31] |
| Graphene composite foam | Domain transformation by pressure | No | 8.0 | ~ 240 | Yes | Yes | 1.2 mm thickness | 2 | [32] |
| Azobenzene polymer | Chain configuration by light | Yes | 3.5 | 10 | No | Yes | 280 nm thickness | 6 | [33] |
| Liquid crystal polymer | Chain configuration by magnetic field | Yes | 1.54 | 600 | Yes | Yes | / | / | [34] |
| Tandem-repeat protein | Chain configuration by hydration | Yes | 2.5 | 20 | No | Yes | ~ 100 µm thickness | 1 | [35] |
| PE nanofiber | Chain configuration by temperature | Yes | 8.0 | / | No | / | 105 nm diameter | / | [36] |
| PE film | Chain configuration by thermal drawing | Yes | 163 | / | No | No | 150 µm thickness<br>~ 10 cm length | / | [4] |
| ELST | Chain configuration by strain and temperature modulation | Yes | 11.5 | 9.4 | Yes | Yes | ~ 1 mm thickness<br>~ 1 cm length | 1000 | This work |

**Table S2. SAXS/WAXS experimental parameters.**

| **Configuration** | **SAXS** | **WAXS** | **WAXS** |
|---|---|---|---|
| Measurement | All | Crack tip | All else |
| Distance from detector (mm) | 1059.1 mm | 109.1 mm | 109.1 mm |
| Aperture Slit Height | 0.7 mm | 0.7 mm | 0.9 mm |
| Aperture Slit Width | 0.3 mm | 0.3 mm | 0.9 mm |
| Wavelength (Å) | 1.5409 ($CuK_{\alpha 1}$) | 1.5409 ($CuK_{\alpha 1}$) | 1.5409 ($CuK_{\alpha 1}$) |
| Power | 45 kV, 0.65 mA | 45 kV, 0.65 mA | 45 kV, 0.65 mA |
| Measurement time (s) | 180 | 300 | 180 |

**Table S3. X-ray characterization of structural parameters for revealing strain effect in the two-way thermal transport mechanism**

| Temperature *T* (°C) | Stretch $\lambda$ | Crystallinity $\chi$ (%) | Hermans orientation factor $f_2$ | Start-to-start spacing *L* (nm) | Crystalline size *D* (nm) |
|---|---|---|---|---|---|
| 25 | 1 | 49.11 | 0.0050 | 11.9 | 10.7 |
| 25 | 5 | 49.95 | 0.8920 | 11.55 | 10.8 |
| 25 | 10 | 53.75 | 0.9074 | 15.17 | 10.98 |
| 25 | 15 | 55.94 | 0.9113 | 15.53 | 11.03 |
| 25 | 20 | 67.07 | 1 | 16.32 | 11.38 |
| 55 | 1 | 1.20 | 0.0020 | / | / |
| 55 | 5 | 1.03 | 0.0020 | / | / |
| 55 | 8 | 30.62 | 0.9705 | / | / |
| 55 | 10 | 32.86 | 0.9291 | 20.5 | 11.76 |
| 55 | 12 | 38.02 | 0.9941 | / | / |
| 55 | 15 | 38.19 | 0.9409 | 20.42 | 11.95 |
| 55 | 18 | 48 | 0.9941 | / | / |
| 55 | 20 | 56.09 | 0.9971 | 23.23 | 11.91 |

**Table S4. X-ray characterization of structural parameters for revealing thermal effect in the two-way thermal transport mechanism**

| Stretch $\lambda$ | Temperature $T$ (°C) | Crystallinity $\chi$ (%) | Hermans' orientation factor $f_2$ | Start-to-start spacing $L$ (nm) | Crystalline size $D$ (nm) |
|---|---|---|---|---|---|
| 1 | 25 | 48.95 | 0 | 11.9 | 10.7 |
| 1 | 30 | 53.87 | 0 | 12.6 | 10.66 |
| 1 | 35 | 52.28 | 0 | 13.4 | 10.82 |
| 1 | 40 | 52.35 | 0 | 14 | 10.92 |
| 1 | 45 | 47.68 | 0 | 15 | 11.15 |
| 1 | 50 | 18.03 | 0 | 23.7 | 11.27 |
| 1 | 55 | 0.34 | 0 | / | / |
| 1 | 60 | 0.27 | 0 | / | / |
| 1 | 65 | 0.42 | 0 | / | / |
| 20 | 25 | 67.24 | 0.9998 | 16.32 | 11.38 |
| 20 | 30 | 65.99 | 0.9935 | 16.5 | 11.06 |
| 20 | 35 | 65.40 | 0.9938 | 16.1 | 11.12 |
| 20 | 40 | 64.37 | 0.9934 | 16.1 | 11.04 |
| 20 | 45 | 62.57 | 0.9938 | 16.3 | 11.51 |
| 20 | 50 | 58.24 | 0.9939 | 19 | 11.42 |
| 20 | 55 | 56.04 | 0.9926 | 23.23 | 11.91 |
| 20 | 60 | 39.18 | 0.9946 | 26.2 | 11.63 |
| 20 | 65 | 29.44 | 0.9939 | 27.3 | 11.03 |

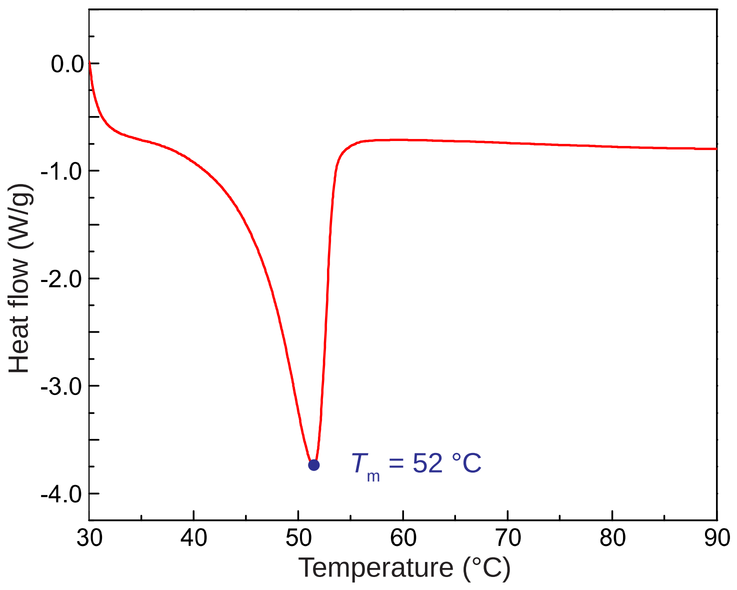


**Figure S1. Differential scanning calorimetry curve of the 20,000 MW ELST.** Endothermic process of melting crystalline domains with the melting point of $T_m$ = 52 °C.

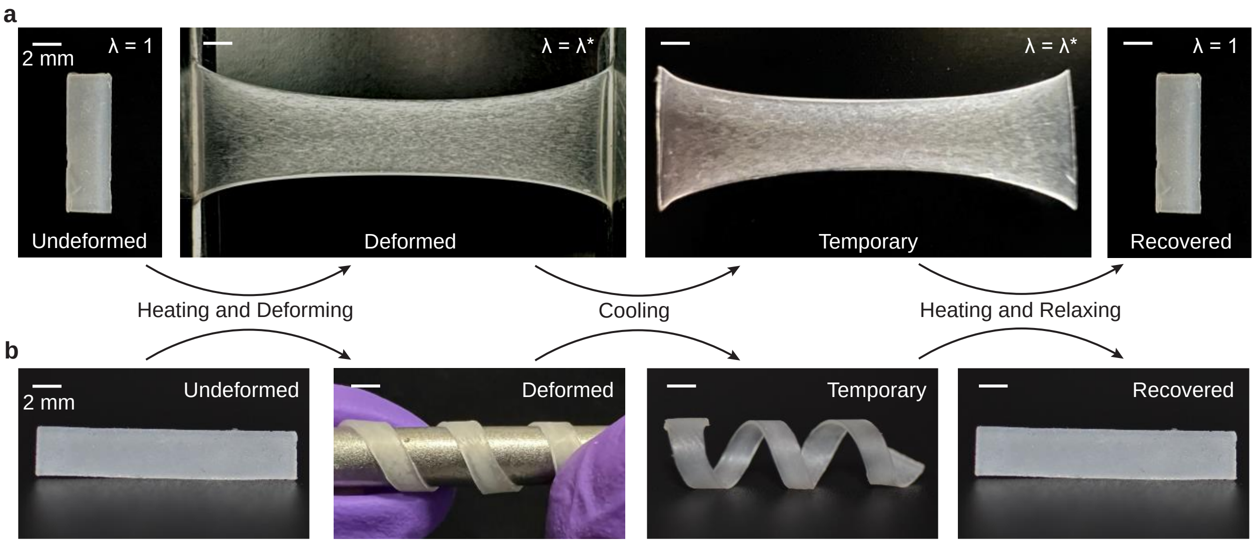


**Figure S2. Images of the ELST under strain and temperature modulations. a)** Dog-bone shaped sample. **b)** Strip sample. Scale bars **a)** and **b)** are 2 mm.

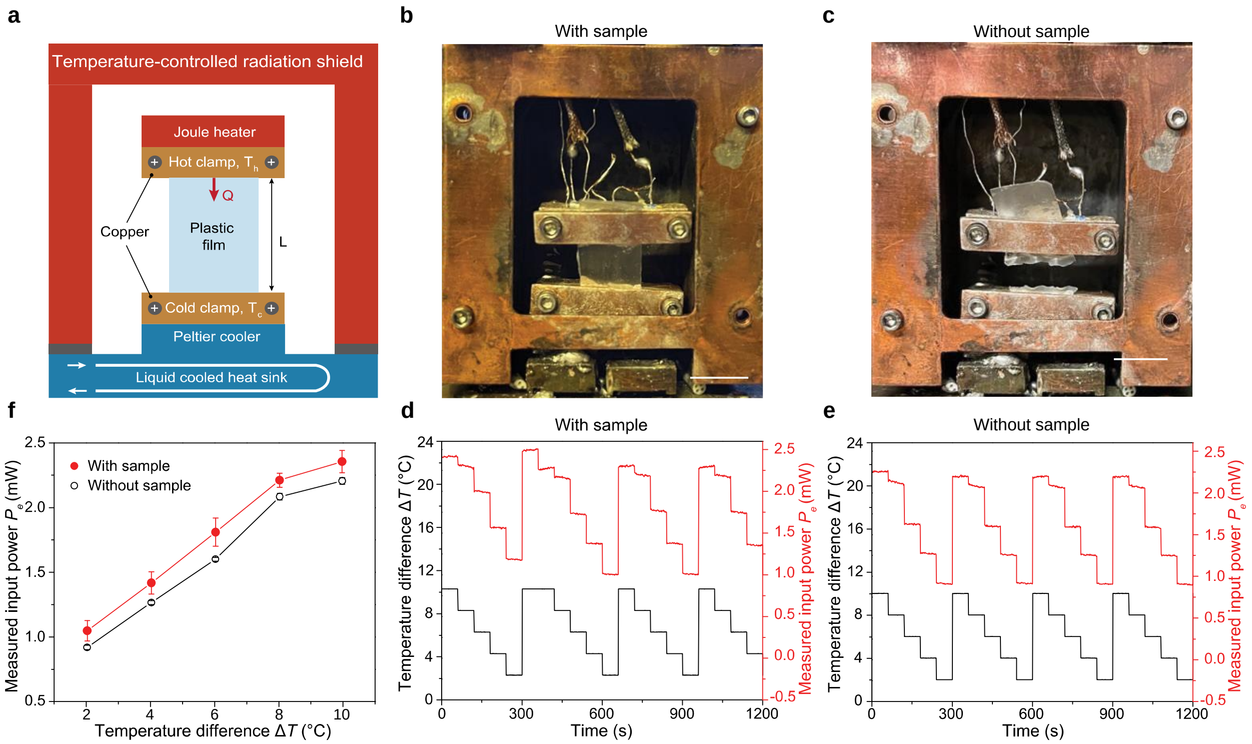


**Figure S3. Steady-state thermal conductivity measurements. a)** Schematic of the home-built steady-state thermal conductivity measurement system. A small temperature difference ($T_h$-$T_c$) across a film sample was created and maintained using Joule heating and thermoelectric cooling inside a high vacuum chamber. **b)** Image of the testing setup with sample. **c)** Image of the testing setup without sample. **d)** Measured input power $P_e$ and temperature difference $\Delta T$ as a function of time for the case with sample. **e)** Measured input power $P_e$ and temperature difference $\Delta T$ as a function of time for the case without sample. **f)** Summarized input power $P_e$ as a function of temperature difference $\Delta T$ with sample and without sample, the slope of which measures $kwt/L$. Scale bars in **b)** and **c)** are 5 mm. Values in **f)** represent the mean and standard deviation ($n$ = 3).

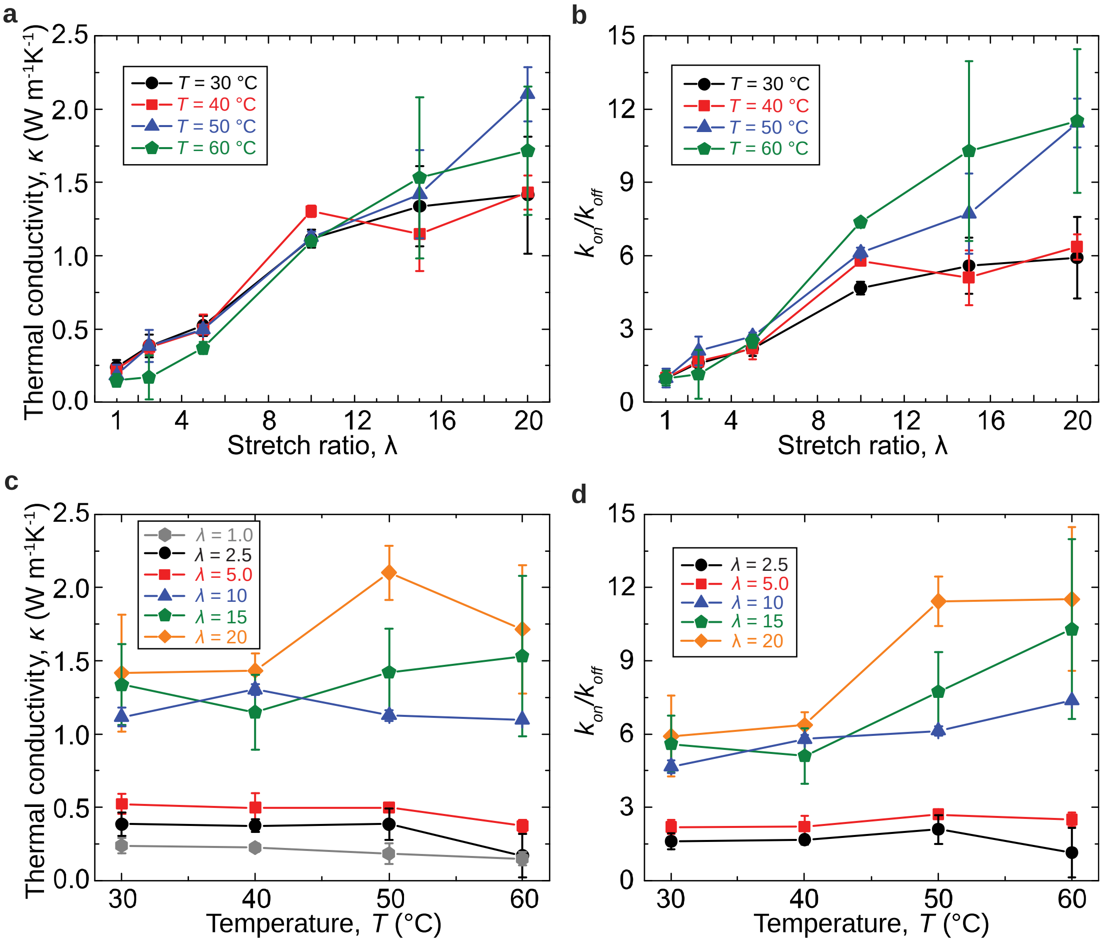


**Figure S4. Steady-state thermal conductivity measurements of the 20,000 MW ELST with various stretch ratios at different temperatures. a)** Thermal conductivity κ and **b)** Thermal conductivity on/off tuning ratio $\kappa_{on}/\kappa_{off}$ versus stretch ratio λ at the temperature of 30, 40, 50, and 60 °C. **c)** Thermal conductivity κ and **d)** thermal conductivity on/off tuning ratio $\kappa_{on}/\kappa_{off}$ versus temperature at various stretch ratios. Values represent the mean and standard deviation ($n$ = 3).

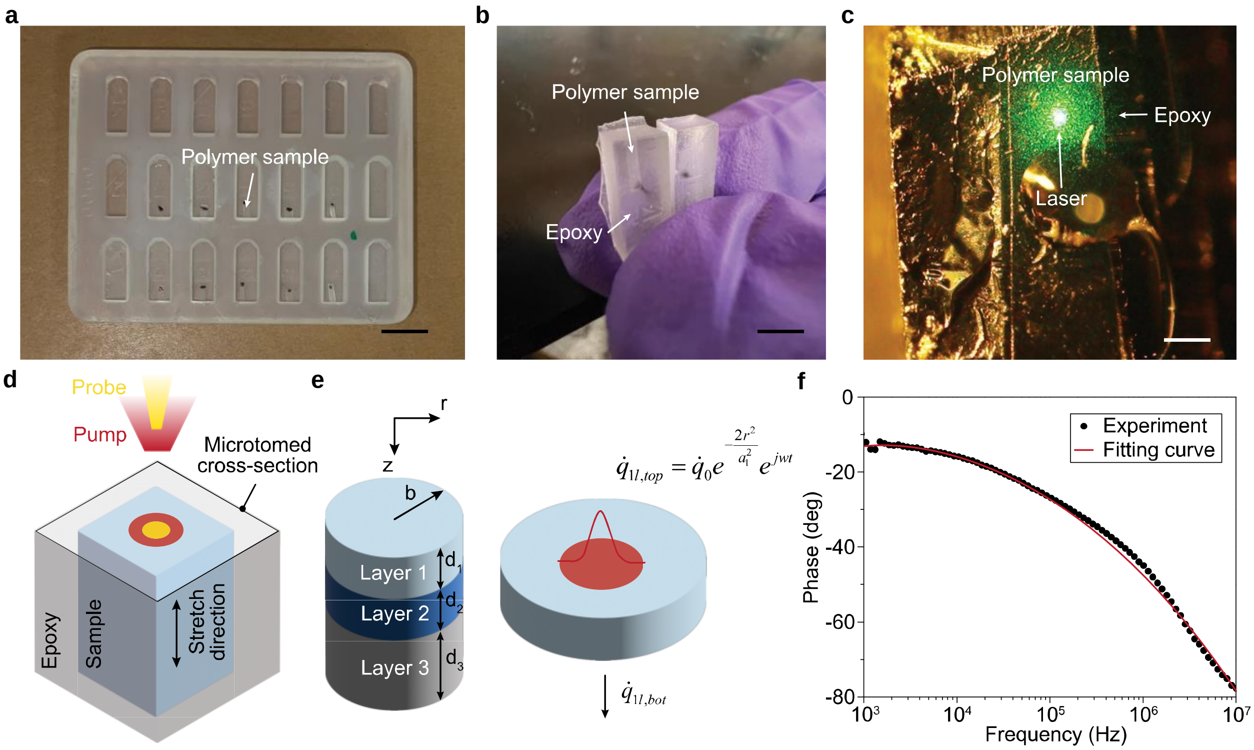


**Figure S5. FDTR thermal conductivity measurements. a)** Image of the mold for curing epoxy resin with the sample embedded. **b)** Image of the cured epoxy resin with embedded sample before and after microtome cutting. **c)** Microscope image of the sample and epoxy on the FDTR platform subject to laser spot. **d)** Schematic illustration of FDTR setup for measuring thermal conductivity. **e)** Analytical model for identifying thermal conductivity of tetra-PEG thermosets. **f)** Representative data and corresponding fitting curve in the plot of phase angle and frequency. Scale bars in **a)**, **b),** and **c)** are 10 mm, 10 mm, 100 μm.

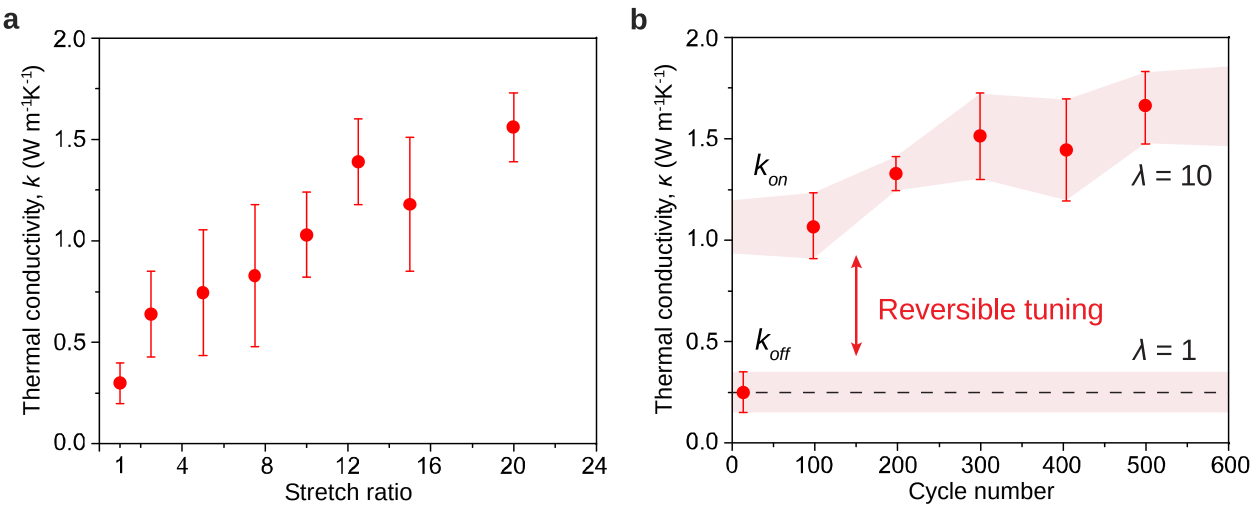


**Figure S6. FDTR thermal conductivity measurements of the 20,000 MW ELST. a)** Thermal conductivity $\kappa$ versus stretch ratio $\lambda$ at the temperature of 25 °C. **b)** Reversible tuning of thermal conductivity by cyclic stretch between $\lambda$ = 1 and $\lambda$ = 10 at the temperature of 25 °C. Values represent the mean and standard deviation (*n* = 3).

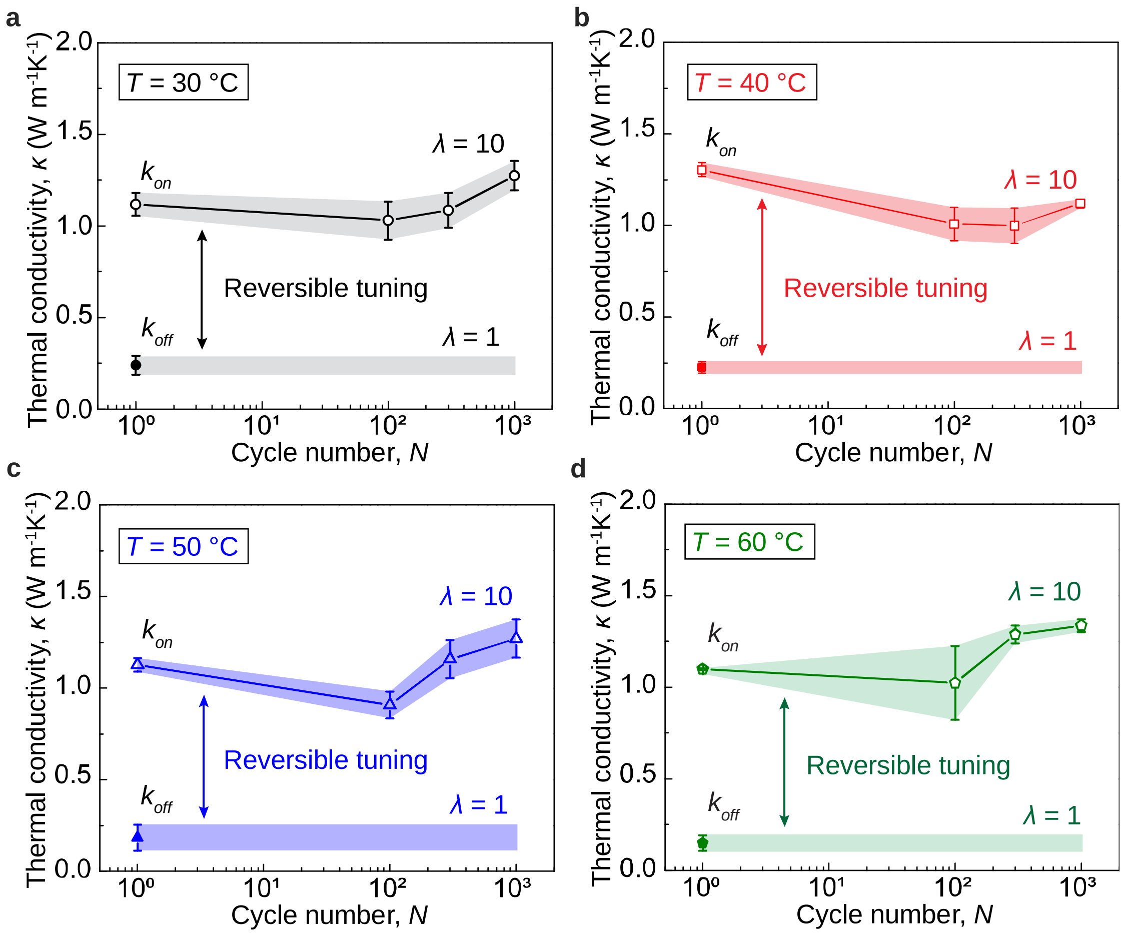


**Figure S7. Steady-state thermal conductivity measurements of the 20,000 MW ELST under cyclic loading.** Reversible tuning of thermal conductivity by cyclic stretch between $\lambda = 1$ and $\lambda = 10$ at the temperature of **a)** 30 °C, **b)** 40 °C, **c)** 50 °C, and **d)** 60 °C. Values represent the mean and standard deviation ($n = 3$).

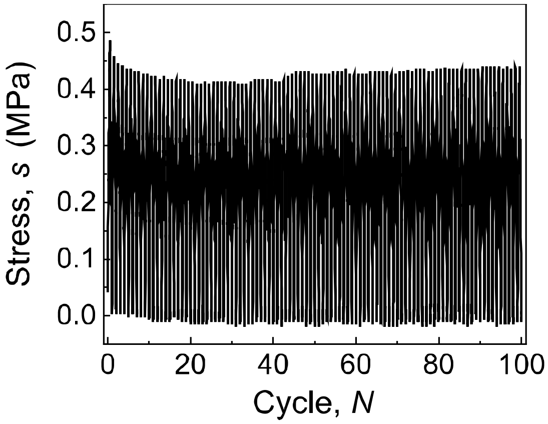


**Figure S8. Maximum stress versus cycle number of the 20,000 MW ELST.** Maximum stress reaches a steady state value as cycle number reaches around 20.

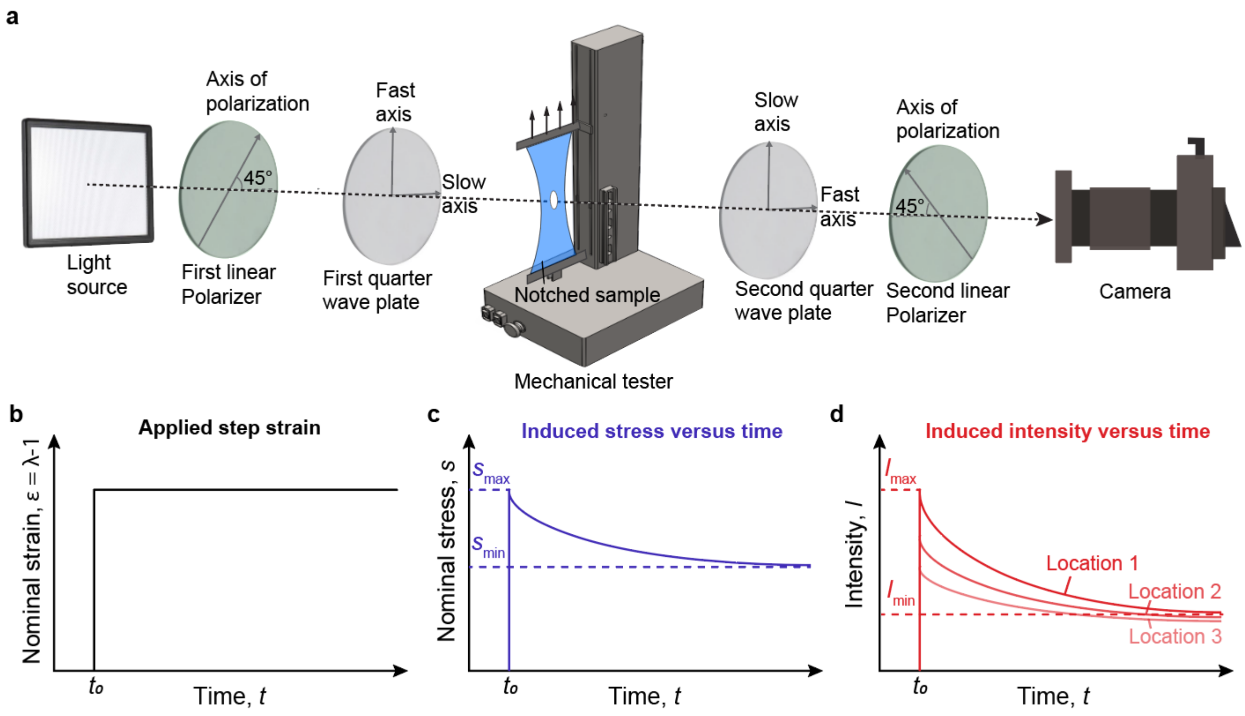


**Figure S9. Design of photoelasticimetry experiments for in-situ structural characterizations. a)** Schematic illustration of the test setup for photoelasticimetry experiments, which contains one light source, one camera, two linear polarizers, and two quarter wave plates. A crack is introduced at the center of the sample to amplify stress levels for enhancing light intensity. **b)** The material is subjected to an applied step strain. **c)** The recorded normal stress of the entire material $s$ as a function of time $t$ for measuring the mean response time of the structural change of the entire material $\tau_m$. **d)** The measured intensity at specific locations on the material $I$ as a function of time for measuring the response time for the structural change at specific locations $\tau$.

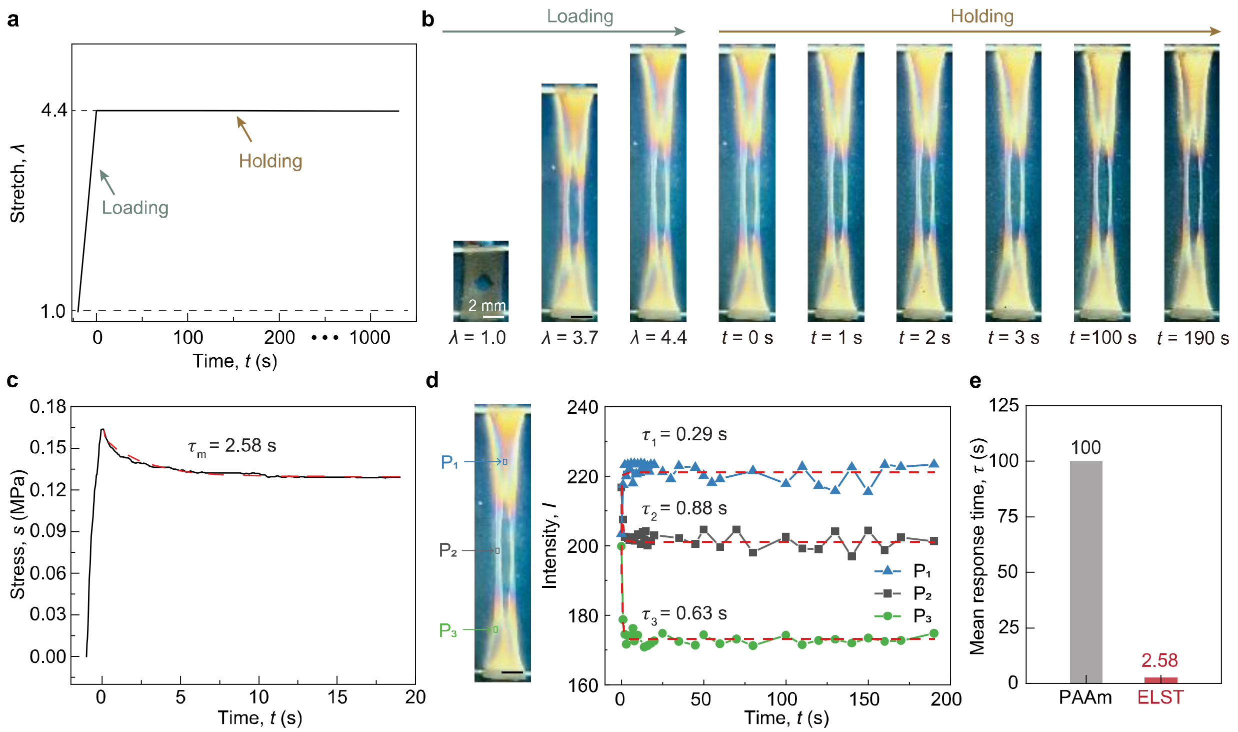


**Figure S10. Relaxation photoelasticimetry experiments. a)** Schematic illustration of an instantaneous stretch applied on the sample followed by stress relaxation. **b)** Image sequences of fringe patterns in our ELST subjected to an instantaneous stretch $\lambda$ of 4.4 along the horizontal direction followed by stress relaxation for up to 200 s. **c)** Measured stress as a function of time fitted into the Voigt model to extract the mean response time for the structural change of the entire material, $\tau_m$ = 2.58 s. **d)** Measured intensities at specific locations $P_1$, $P_2$ and $P_3$ as a function of time fitted into the Voigt model to extract the corresponding response time for the structural change at these locations $\tau_1$ = 0.29 s, $\tau_2$ = 0.88 s, and $\tau_3$ = 0.63 s. **e)** Comparison of the mean response time $\tau_m$ between PAAm gel and our ELST. Scale bars in **b)** and **d)** are 2 mm.

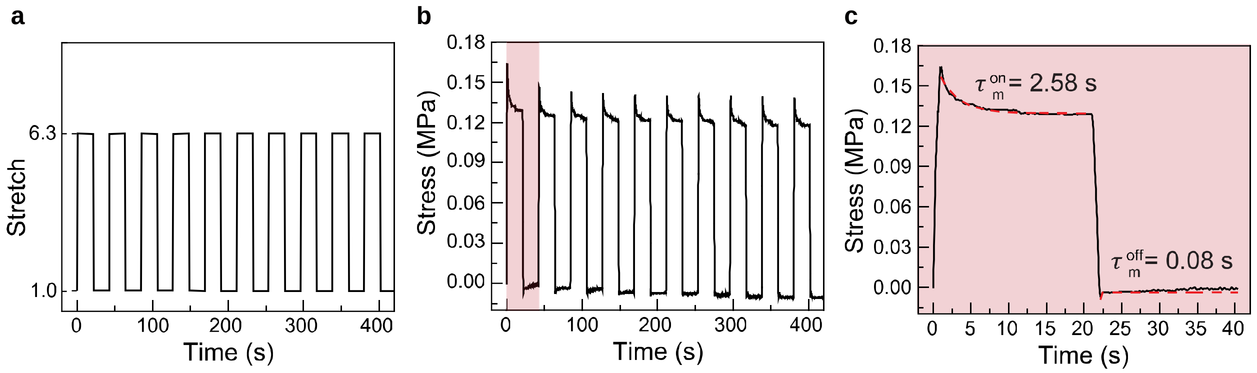


**Figure S11. Characterization of on-off response time. a)** The material is subjected to cyclic step stretch. **b)** Measured stress as a function of time under cyclic loading. **c)** Measured stress as a function of time under one cycle of loading to extract the on and off response time as 2.58 s and 0.08 s, respectively.

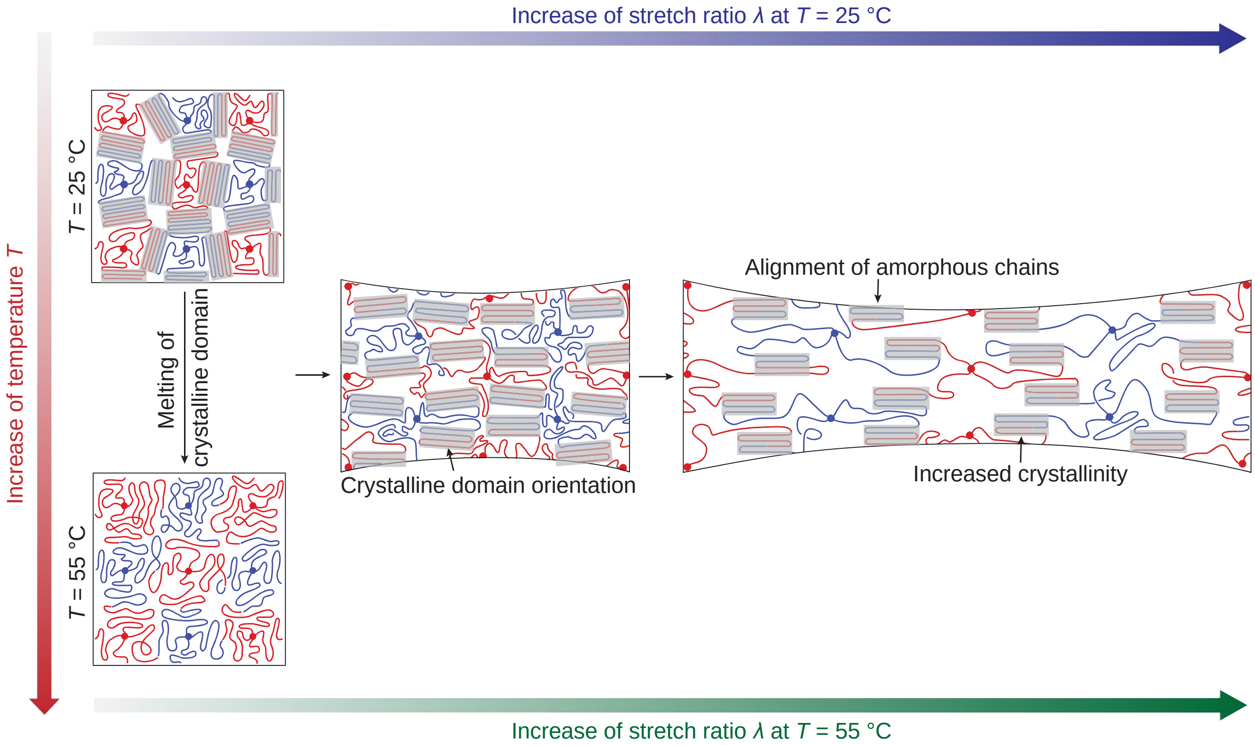


**Figure S12. Schematic illustration of the two-way thermal conductivity tuning mechanism.** The applied mechanical strain promotes phonon transport due to the synergy of oriented crystalline domains, aligned interstitial amorphous chains, and increased crystallinity. The increased temperature induces the hinderance of phonon transport due to the crystalline-to-amorphous transition and the augmentation of phonon transport due to the aligned polymer chains, given their increased flexibility.

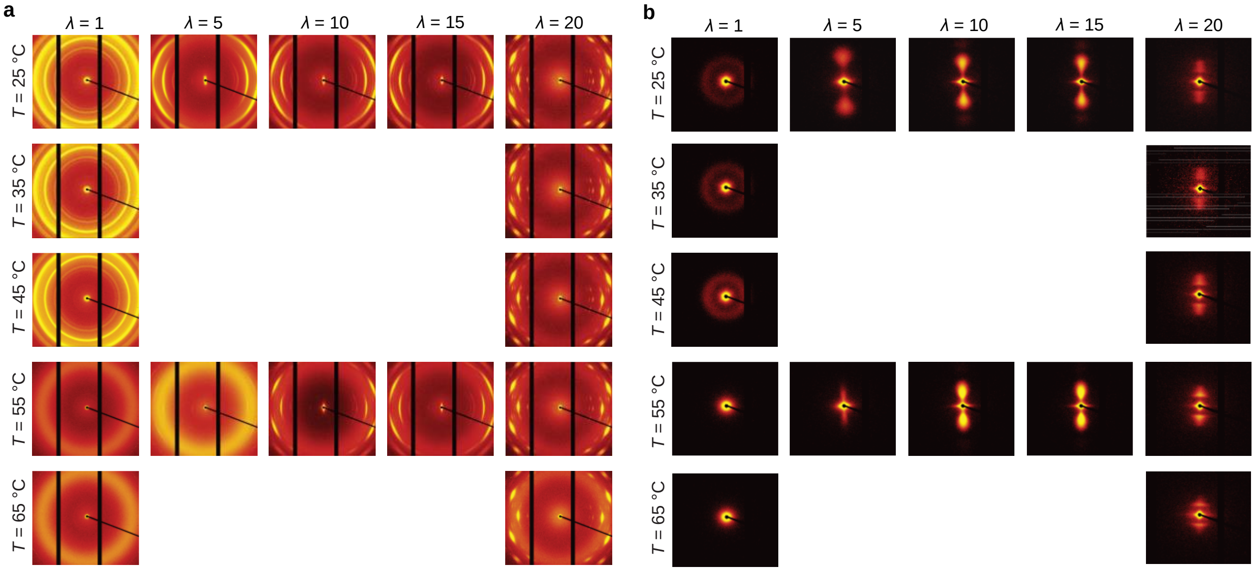


**Figure S13. X-ray characterizations of ELST subjected to controlled strain and temperature variations. a)** WAXS scattering patterns. **b)** SAXS scattering patterns.

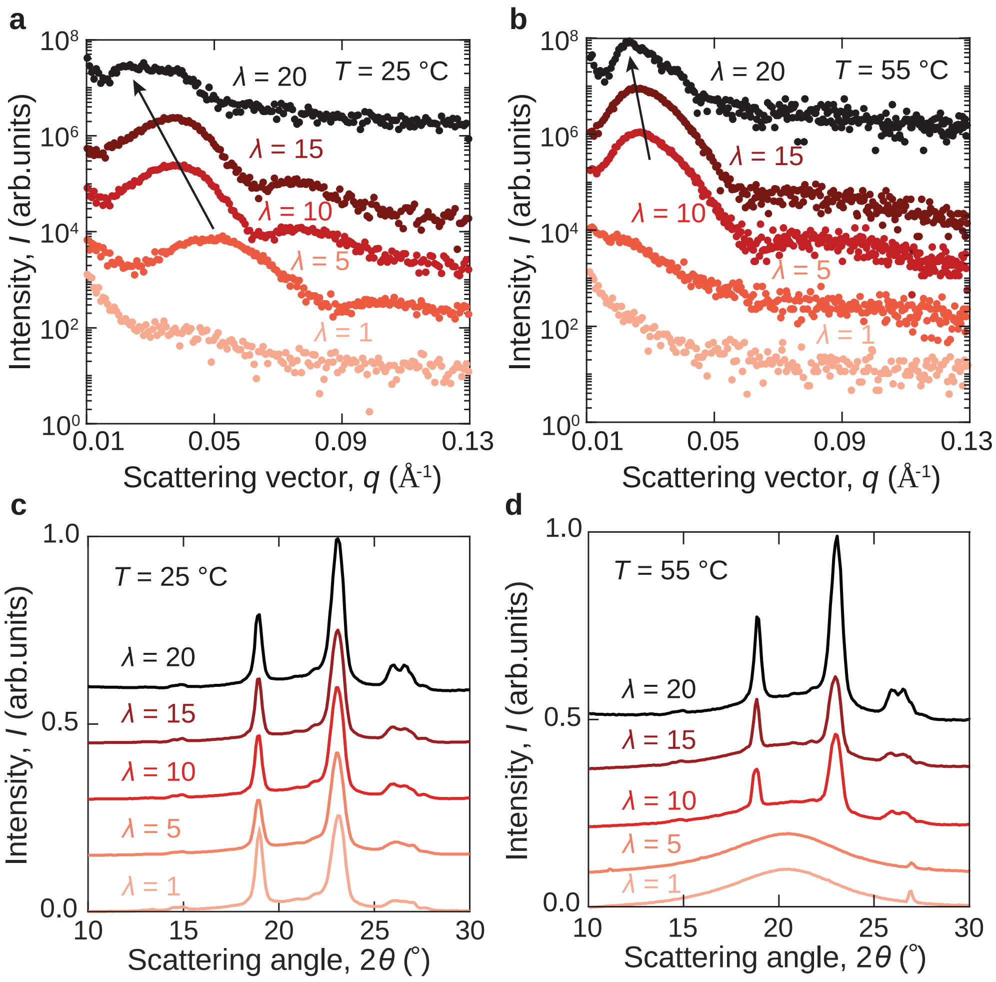


**Figure S14. X-ray scattering intensity profiles of the 20,000 MW ELST at different stretch ratios. a)** SAXS of ELST at *T* = 25 °C, **b)** SAXS of ELST at *T* = 55 °C, **c)** WAXS of ELST at *T* = 25 °C, **d)** WAXS of ELST at *T* = 55 °C.

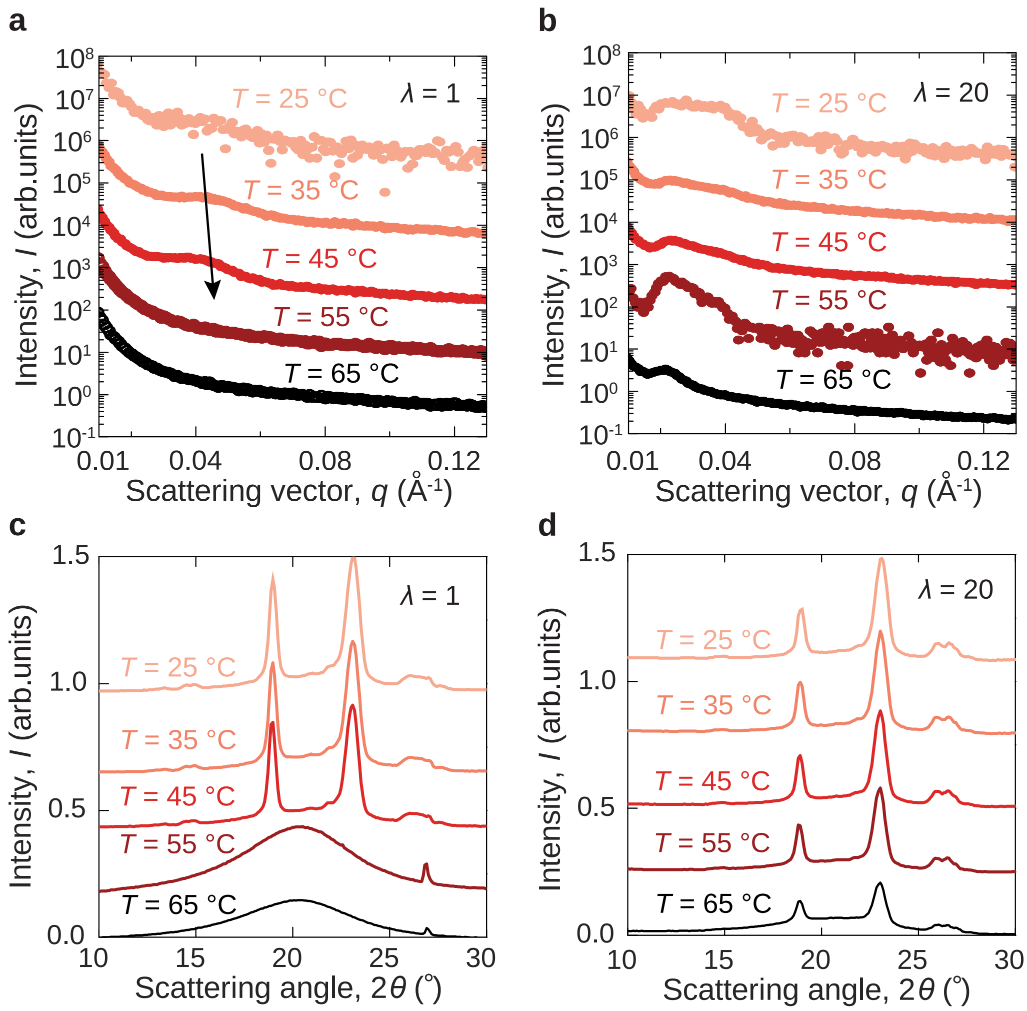


**Figure S15. X-ray scattering intensity profiles of the 20,000 MW ELST at different temperatures. a)** SAXS of ELST at undeformed state (i.e., $\lambda$ = 1), **b)** SAXS of ELST at highly deformed state (i.e., $\lambda$ = 20), **c)** SAXS of ELST at undeformed state (i.e., $\lambda$ = 1), **d)** SAXS of ELST at highly deformed state (i.e., $\lambda$ = 20).

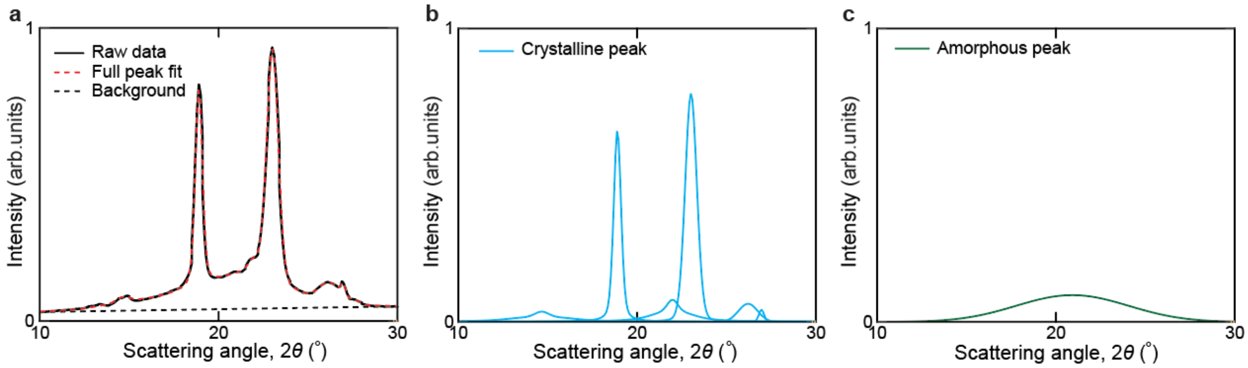


**Figure S16. Decomposition of 1D WAXS profile. a)** Full peak fit of raw data of 1D WAXS profile, **b)** extracted crystalline peak, **c)** extracted amorphous peak.

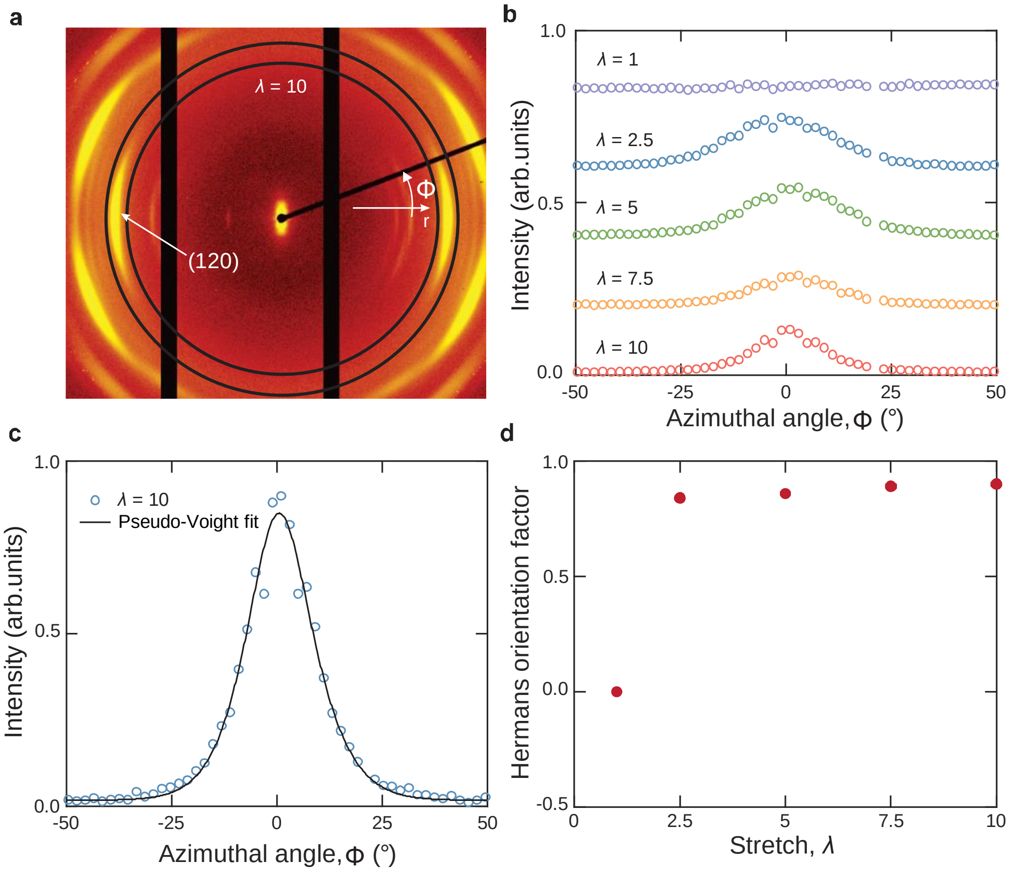


**Figure S17. Identification of Hermans' orientation factor. a)** Scattering pattern at stretch ratio of 10, **b)** Scattering intensity as a function of Azimuthal angle at various stretches, **c)** Pseudo-Voight fit of scattering intensity versus Azimuthal angle, **d)** Calculated Hermans orientation factor.

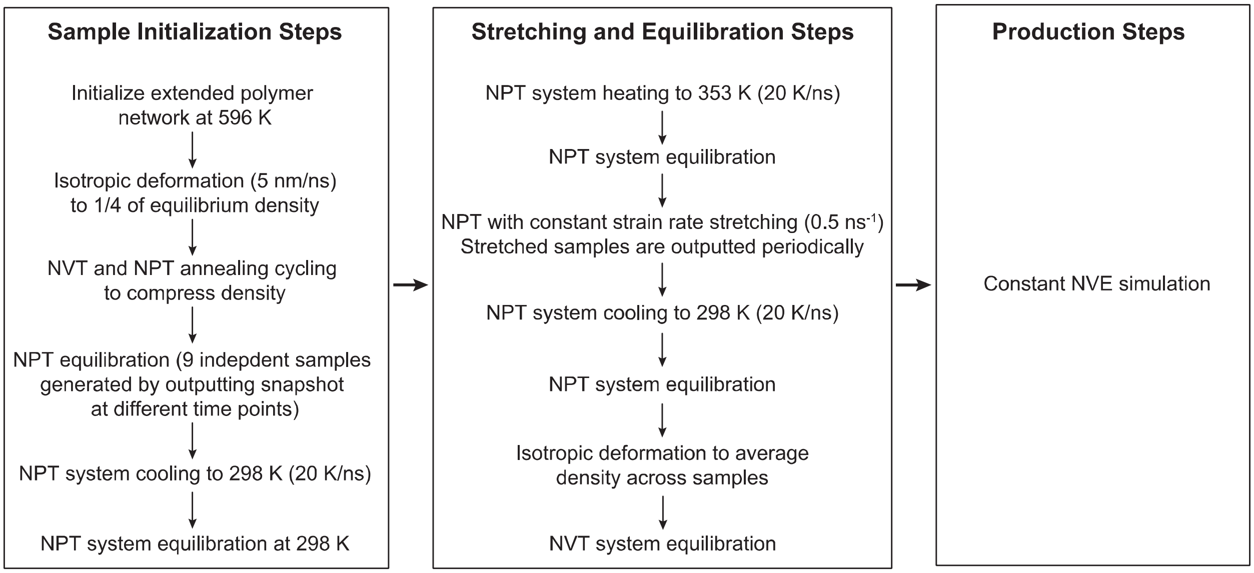


**Figure S18. Molecular dynamics simulation setup procedures.** Sample initialization steps, stretching and equilibration steps, and production steps to generate samples for structure and thermal conductivity calculations.

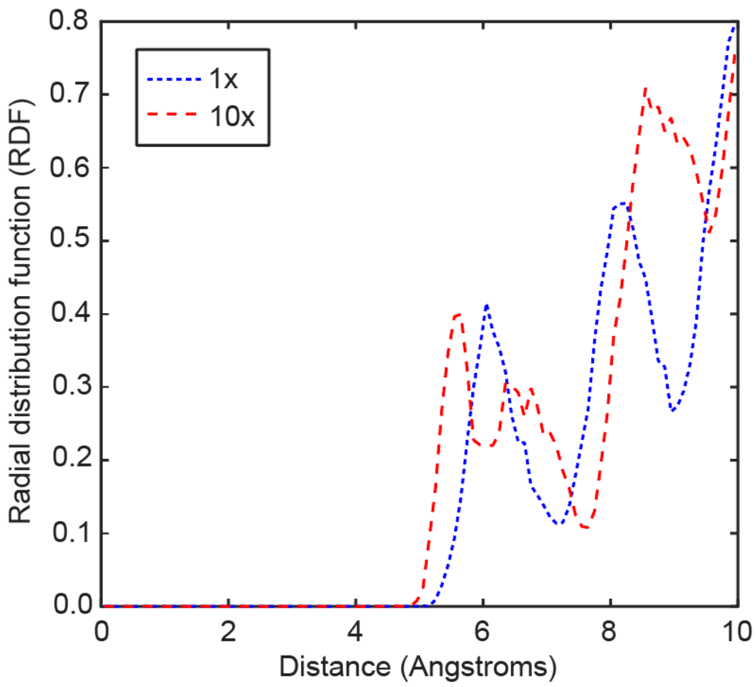


**Figure S19. Snapshots and structural analysis of the simulated 10,000 MW tetra-PEG thermosets.** Radial distribution function of the crosslinked carbon atoms in the unstretched ($\lambda = 1$) and stretched $\lambda = 10$ samples.

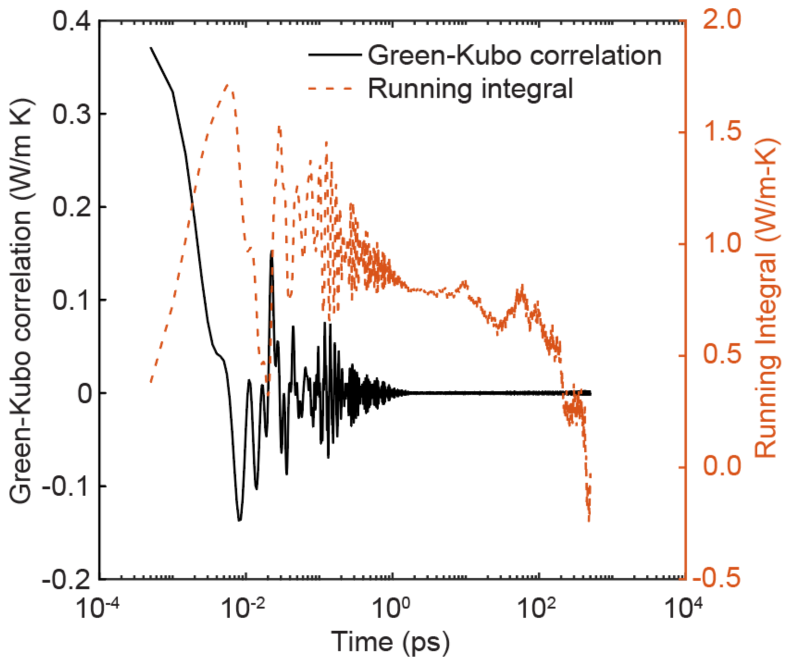


**Figure S20. Thermal conductivity measurement in simulation.** Representative Green-Kubo correlation and running integral for sample stretched to $\lambda = 6$.

## References


1. Lin, S., et al., *Fracture and fatigue of ideal polymer networks.* Extreme Mechanics Letters, 2021. **48**: p. 101399.
2. Akagi, Y., et al., *Fracture energy of polymer gels with controlled network structures.* The Journal of Chemical Physics, 2013. **139**(14): p. 144905.
3. Kraemer, D. and G. Chen, *A simple differential steady-state method to measure the thermal conductivity of solid bulk materials with high accuracy.* Review of Scientific Instruments, 2014. **85**(2): p. 025108.
4. Xu, Y., et al., *Nanostructured polymer films with metal-like thermal conductivity.* Nature Communications, 2019. **10**(1): p. 1-8.
5. Yang, J., C. Maragliano, and A.J. Schmidt, *Thermal property microscopy with frequency domain thermoreflectance.* Review of Scientific Instruments, 2013. **84**(10): p. 104904.
6. Yang, J., E. Ziade, and A.J. Schmidt, *Uncertainty analysis of thermoreflectance measurements.* Review of Scientific Instruments, 2016. **87**(1): p. 014901.
7. Schmidt, A.J., R. Cheaito, and M. Chiesa, *Characterization of thin metal films via frequency-domain thermoreflectance.* Journal of Applied Physics, 2010. **107**(2): p. 024908.
8. Schmidt, A.J., R. Cheaito, and M. Chiesa, *A frequency-domain thermoreflectance method for the characterization of thermal properties.* Review of Scientific Instruments, 2009. **80**(9): p. 094901.
9. Kou, Y., et al., *Thermal analysis and heat capacity study of polyethylene glycol (PEG) phase change materials for thermal energy storage applications.* The Journal of Chemical Thermodynamics, 2019. **128**: p. 259-274.
10. Rajagopal, K.R., *A note on a reappraisal and generalization of the Kelvin–Voigt model.* Mechanics Research Communications, 2009. **36**(2): p. 232-235.
11. Tadokoro, H., et al., *Structural studies on polyethers,[-(CH2) m-O-] n. II. Molecular structure of polyethylene oxide.* Die Makromolekulare Chemie: Macromolecular Chemistry and Physics, 1964. **73**(1): p. 109-127.
12. Takahashi, Y., I. Sumita, and H. Tadokoro, *Structural studies of polyethers. IX. Planar zigzag modification of poly (ethylene oxide).* Journal of Polymer Science: Polymer Physics Edition, 1973. **11**(11): p. 2113-2122.
13. Takahashi, Y. and H. Tadokoro, *Structural studies of polyethers,(-(CH2) mO-) n. X. Crystal structure of poly (ethylene oxide).* Macromolecules, 1973. **6**(5): p. 672-675.
14. Wilchinsky, Z.W., *Measurement of orientation in polypropylene film.* Journal of Applied Physics, 1960. **31**(11): p. 1969-1972.
15. Zsigmondy, R. and P. Scherrer, *Bestimmung der inneren Struktur und der Größe von Kolloidteilchen mittels Röntgenstrahlen.* Kolloidchemie Ein Lehrbuch, 1912: p. 387-409.
16. Sun, H., *COMPASS: an ab initio force-field optimized for condensed-phase applications overview with details on alkane and benzene compounds.* The Journal of Physical Chemistry B, 1998. **102**(38): p. 7338-7364.
17. Rigby, D., H. Sun, and B. Eichinger, *Computer simulations of poly (ethylene oxide): force field, pvt diagram and cyclization behaviour.* Polymer International, 1997. **44**(3): p. 311-330.
18. Boone, P., H. Babaei, and C.E. Wilmer, *Heat flux for many-body interactions: corrections to LAMMPS.* Journal of Chemical Theory and Computation, 2019. **15**(10): p. 5579-5587.
19. Surblys, D., et al., *Application of atomic stress to compute heat flux via molecular dynamics for systems with many-body interactions.* Physical Review E, 2019. **99**(5): p. 051301.

20. Surblys, D., et al., *Methodology and meaning of computing heat flux via atomic stress in systems with constraint dynamics.* Journal of Applied Physics, 2021. **130**(21): p. 215104.
21. Lu, Q., et al., *Bi-directional tuning of thermal transport in SrCoOx with electrochemically induced phase transitions.* Nature Materials, 2020. **19**(6): p. 655-662.
22. Sood, A., et al., *An electrochemical thermal transistor.* Nature Communications, 2018. **9**(1): p. 1-9.
23. Cho, J., et al., *Electrochemically tunable thermal conductivity of lithium cobalt oxide.* Nature Communications, 2014. **5**(1): p. 1-6.
24. Zhang, Z.-T., et al., *Tuning the thermal conductivity of nanoparticle suspensions by electric field.* Nanotechnology, 2020. **31**(46): p. 465403.
25. Sun, P., et al., *Magnetic graphite suspensions with reversible thermal conductivity.* Materials Letters, 2015. **149**: p. 92-94.
26. Lee, S., et al., *Anomalously low electronic thermal conductivity in metallic vanadium dioxide.* Science, 2017. **355**(6323): p. 371-374.
27. Zheng, R., et al., *Reversible temperature regulation of electrical and thermal conductivity using liquid–solid phase transitions.* Nature Communications, 2011. **2**(1): p. 1-6.
28. Sun, P., et al., *Room temperature electrical and thermal switching CNT/hexadecane composites.* Advanced Materials, 2013. **25**(35): p. 4938-4943.
29. Foley, B.M., et al., *Voltage-controlled bistable thermal conductivity in suspended ferroelectric thin-film membranes.* ACS Applied Materials & Interfaces, 2018. **10**(30): p. 25493-25501.
30. Yang, F., et al., *Large magnetoresistance of electrodeposited single-crystal bismuth thin films.* Science, 1999. **284**(5418): p. 1335-1337.
31. Li, M., et al., *Electrically gated molecular thermal switch.* Science, 2023. **382**(6670): p. 585-589.
32. Du, T., et al., *Wide range continuously tunable and fast thermal switching based on compressible graphene composite foams.* Nature Communications, 2021. **12**(1): p. 1-10.
33. Shin, J., et al., *Light-triggered thermal conductivity switching in azobenzene polymers.* Proceedings of the National Academy of Sciences, 2019. **116**(13): p. 5973-5978.
34. Shin, J., et al., *Thermally functional liquid crystal networks by magnetic field driven molecular orientation.* ACS Macro Letters, 2016. **5**(8): p. 955-960.
35. Tomko, J.A., et al., *Tunable thermal transport and reversible thermal conductivity switching in topologically networked bio-inspired materials.* Nature Nanotechnology, 2018. **13**(10): p. 959-964.
36. Shrestha, R., et al., *High-contrast and reversible polymer thermal regulator by structural phase transition.* Science Advances, 2019. **5**(12): p. eaax3777.
